# Limited utility of tissue micro-arrays in detecting intra-tumoral heterogeneity in stem cell characteristics and tumor progression markers in breast cancer

**DOI:** 10.1186/s12967-018-1495-6

**Published:** 2018-05-08

**Authors:** Pascale Kündig, Charlotte Giesen, Hartland Jackson, Bernd Bodenmiller, Bärbel Papassotirolopus, Sandra Nicole Freiberger, Catharine Aquino, Lennart Opitz, Zsuzsanna Varga

**Affiliations:** 1 Institute of Pathology and Molecular Pathology, University Hospital Zurich, Schmelzbergstrasse 12, 8091 Zurich, Switzerland; 2 Institute of Molecular Life Sciences, University of Zurich, Zurich, Switzerland; 3 Breast Center Seefeld, Zurich, Switzerland; 4 Functional Genomics Center Zurich, Zurich, Switzerland

**Keywords:** Intratumoral heterogeneity, Breast cancer, Stem cells, Tumor progression, Tissue micro array

## Abstract

**Background:**

Intra-tumoral heterogeneity has been recently addressed in different types of cancer, including breast cancer. A concept describing the origin of intra-tumoral heterogeneity is the cancer stem-cell hypothesis, proposing the existence of cancer stem cells that can self-renew limitlessly and therefore lead to tumor progression. Clonal evolution in accumulated single cell genomic alterations is a further possible explanation in carcinogenesis. In this study, we addressed the question whether intra-tumoral heterogeneity can be reliably detected in tissue-micro-arrays in breast cancer by comparing expression levels of conventional predictive/prognostic tumor markers, tumor progression markers and stem cell markers between central and peripheral tumor areas.

**Methods:**

We analyzed immunohistochemical expression and/or gene amplification status of conventional prognostic tumor markers (ER, PR, HER2, CK5/6), tumor progression markers (PTEN, PIK3CA, p53, Ki-67) and stem cell markers (mTOR, SOX2, SOX9, SOX10, SLUG, CD44, CD24, TWIST) in 372 tissue-micro-array samples from 72 breast cancer patients. Expression levels were compared between central and peripheral tumor tissue areas and were correlated to histopathological grading. 15 selected cases additionally underwent RNA sequencing for transcriptome analysis.

**Results:**

No significant difference in any of the analyzed between central and peripheral tumor areas was seen with any of the analyzed methods/or results that showed difference. Except mTOR, PIK3CA and SOX9 (nuclear) protein expression, all markers correlated significantly (p < 0.05) with histopathological grading both in central and peripheral areas.

**Conclusion:**

Our results suggest that intra-tumoral heterogeneity of stem-cell and tumor-progression markers cannot be reliably addressed in tissue-micro-array samples in breast cancer. However, most markers correlated strongly with histopathological grading confirming prognostic information as expression profiles were independent on the site of the biopsy was taken.

**Electronic supplementary material:**

The online version of this article (10.1186/s12967-018-1495-6) contains supplementary material, which is available to authorized users.

## Background

Different types of cancer, including breast cancer, were found to present intra-tumoral heterogeneity, which can occur as genetic, phenotypic or functional diversity in spatial or temporal patterns [1]. The two most commonly accepted concepts describing the origin of tumor heterogeneity are the cancer stem cell (CSC) hypothesis and the Darwinian clonal evolution model [2, 3]. Both thesis consider gain of proliferative potential through single cells that acquired multiple molecular alterations. The Darwinian clonal evolution model proposes natural selection through varying degrees of genetic instability, leading to multiple subpopulations with different genetic aberrations. On the other hand, the cancer stem cell hypothesis posits the existence of a small population of tumorigenic cells within a tumor, that can self-renew limitlessly and therefore induces tumor growth, disease progression, propensity for metastasis and the generation of heterogeneity [3–5]. Moreover, cancer stem cells lead to therapy resistance [5–7]. In 2003, Al-Hajj et al. first described the presence of cancer stem cells in human breast cancer [6, 8].

When using core needle biopsy or fine-needle aspirations for tumor diagnostics only a small amount of tissue is examined. Therapeutic decisions are based on those single tumor samples. Accordingly, the findings of regional intra-tumoral heterogeneity of biomarkers query the significance of single biopsy interpretations with implications for accurate tumor classification [9–11]. Huebschman et al. previously examined adjacent cells in triple negative breast cancer at which a significant molecular heterogeneity, including as well markers of the “stem cell” type, was found [10]. Allott et al. revealed spatial heterogeneity to be a source of core-to-core discordance in status of estrogen receptor (ER), progesterone receptor (PR), and human epidermal growth factor 2 (HER2) on tissue micro arrays (TMAs) when analyzed using automated digital image. They therefore suggested intra-tumoral heterogeneity to have possible implications for breast cancer classifications [11]. To optimize therapy, molecular biomarkers are required, that exactly predict clinical disease outcomes and can be targets of molecular therapies [12].

In our study, we addressed the question whether intra-tumoral heterogeneity of breast stem cell and tumor progression markers can be reliably detected in TMA based tumor samples by comparing protein and gene expression levels between central and peripheral tumor parts. For this purpose, a special TMA was constructed containing peripheral and central primary breast cancer and as well as available also metastatic samples. Furthermore, we proofed central and peripheral areas whether they bear the same prognostic information regarding stem cell and tumor progression markers on the base of histopathological grading.

## Methods

### Patient cohort

In this study, a special tissue micro array (TMA) was constructed using 372 formalin-fixed, paraffin embedded (FFPE) tissue samples from 72 breast cancer patients. The cohort contained 372 samples from the Institute of Pathology and Molecular Pathology, University Hospital of Zurich, collected between 1998 and 2011. Samples for the TMA were punched from central and peripheral primary tumor tissue areas in all n = 72 cases. The distance between central and peripheral areas was on average 1 cm. Samples from axillary lymph node or skin metastases were available in n = 11 cases. Clinico-pathological data such as histological grading, subtype and tumor/nodal stage and information about patient follow-up could be retrieved from the database of the Institute of Pathology and Molecular Pathology, University Hospital of Zurich. Additional data on patient survival was provided by the Breast Center Seefeld, Zurich.

### Clinico-pathological parameters

In total, 57 of 72 (79%) cases were invasive ductal carcinomas (NST, non-special type) (G1: n = 16, G2: n = 16, G3: n = 25), n = 9 (13%) cases were invasive lobular carcinomas (G1: n = 0, G2: n = 8, G3: n = 1) and n = 5 (7%) tumors were classified as other histological subtypes. Information on histological subtype was missing in n = 1 (1%) case.

Altogether, pT stage was available in 67 of 72 cases (93%). The information was missing for n = 5 (7%) patients. N = 31 (43%) tumors were staged pT1 (G1: n = 12, G2: n = 8, G3: n = 11), n = 26 (36%) tumors were staged pT2 (G1: n = 6, G2: n = 10, G3: n = 10), n = 9 (13%) tumors were staged pT3 (G1: n = 0, G2: n = 5, G3: n = 4) and n = 1 (1%) tumor was classified pT4 (G1: n = 0, G2: n = 0, G3: n = 1).

Nodal stage was available in n = 63 cases. The information was missing in n = 9 (13%) patients. N = 28 (39%) cases were nodal negative (pN0, G1: n = 11, G2: n = 8, G3: n = 9). Of the nodal positive patients, n = 26 (36%) tumors were classified as pN1 (G1: n = 7, G2: n = 7, G3: n = 12), n = 5 (7%) tumors were classified as pN2 (G1: n = 1, G2: n = 3, G3: n = 1) and n = 4 (6%) tumors were staged as pN3 (G1: n = 0, G2: n = 2, G3: n = 3).

Clinico-pathological parameters are summarized in Table 1.

**Table 1:** Clinico-pathological parameters

*n* = *72s*	Histological grading							
	G1		G2		G3		Total	
	n	%	n	%	n	%	n	%
Type of tissue								
Tumor center	19	100	26	100	27	100	72	100
Tumor periphery	19	100	26	100	27	100	72	100
Normal tissue	19	100	26	100	27	100	72	100
Lymph node metastasis	1	5	6	23	4	15	11	15
Skin metastasis	0	0	1	4	0	0	1	1
Total	19	100	26	100	27	100	72	100
Histological subtype								
Invasive ductal	16	84	16	62	25	93	57	79
Invasive lobular	0	0	8	31	1	4	9	13
Other	3	16	2	8	0	0	5	7
Unknown	0	0	0	0	1	4	1	1
Total	19	100	26	100	27	100	72	100
pT stage								
pT1	12	63	8	31	11	41	31	43
pT2	6	32	10	38	10	37	26	36
pT3	0	0	5	19	4	15	9	13
pT4	0	0	0	0	1	4	1	1
Unknown	1	5	3	12	1	4	5	7
Total	19	100	26	100	27	100	72	100
pN stage								
pN0	11	58	8	31	9	33	28	39
pN1	7	37	7	27	12	44	26	36
pN2	1	5	3	12	1	4	5	7
pN3	0	0	2	8	2	7	4	6
Unknown	0	0	6	23	3	11	9	13
Total	19	100	26	100	27	100	72	100

### Tissue micro array (TMA)

Formalin-fixed, paraffin embedded (FFPE) tissue samples from 72 breast cancer patients were arranged into one tissue micro array (TMA 208) using a method of construction previously described [13, 14]. From each patient (n = 72) two tissue cores of central tumor parts, as well as two tissue cores of peripheral tumor parts and at least one core of normal tissue were punched and added to the TMA. Areas to be selected for the TMA were determined on hematoxylin and eosin (HE) stained large tissue sections from routine diagnostic slides. Additionally, in 15% of cases (n = 11) two cores from lymph node metastasis and in 1% of cases (n = 1) two cores of skin metastasis were added to the TMA. Selection of tumor areas were processed for the TMA in the same way as with the primary tumors.

Tissue cores were grouped per patient and according to the histopathological grading [15]. Of 72 cases 19 were well differentiated (G1), n = 26 were moderately differentiated (G2) and n = 27 were poorly differentiated (G3).

### Immunohistochemistry

All immunohistochemical stains were performed in the Immunohistochemistry Laboratory of the Institute of Pathology and Molecular Pathology, University Hospital Zurich, with following clones and pretreatment modalities using the automated Ventana Benchmark stain system (Additional file 1: Table S1).

### Scoring of immunohistochemical stains

The expressions of E-cadherin, p63, CK5/6, CD24, mTOR and PIK3CA were scored with a two-tiered scoring system (negative vs. positive) being membranous and/or nuclear positive.

The expressions of SLUG, SOX2, SOX9, SOX10, PTEN, TWIST, p53 and CD44 were scored with a three-tiered scoring system (negative, ≤ 50% of the cells positive, > 50% of the cells positive), being cytoplasmic and/or nuclear positive.

The membranous expressions of HER2 and EGFR were scored with a four-tiered scoring system (negative, ≤ 10% positive, > 10% but incomplete positive, strong circular positivity in > 10% of the cells), according to current diagnostic guidelines as score 0, 1+, 2+, 3+ [16].

The nuclear expressions of ER, PR and Ki-67 were scored with a scoring system representing the estimated proportion of positive tumor cells (negative, 10% positive, 20% positive, 30% positive, 40% positive, 50% positive, 60% positive, 70% positive, 80% positive, 90% positive, 100% positive).

**Fig. 1:**
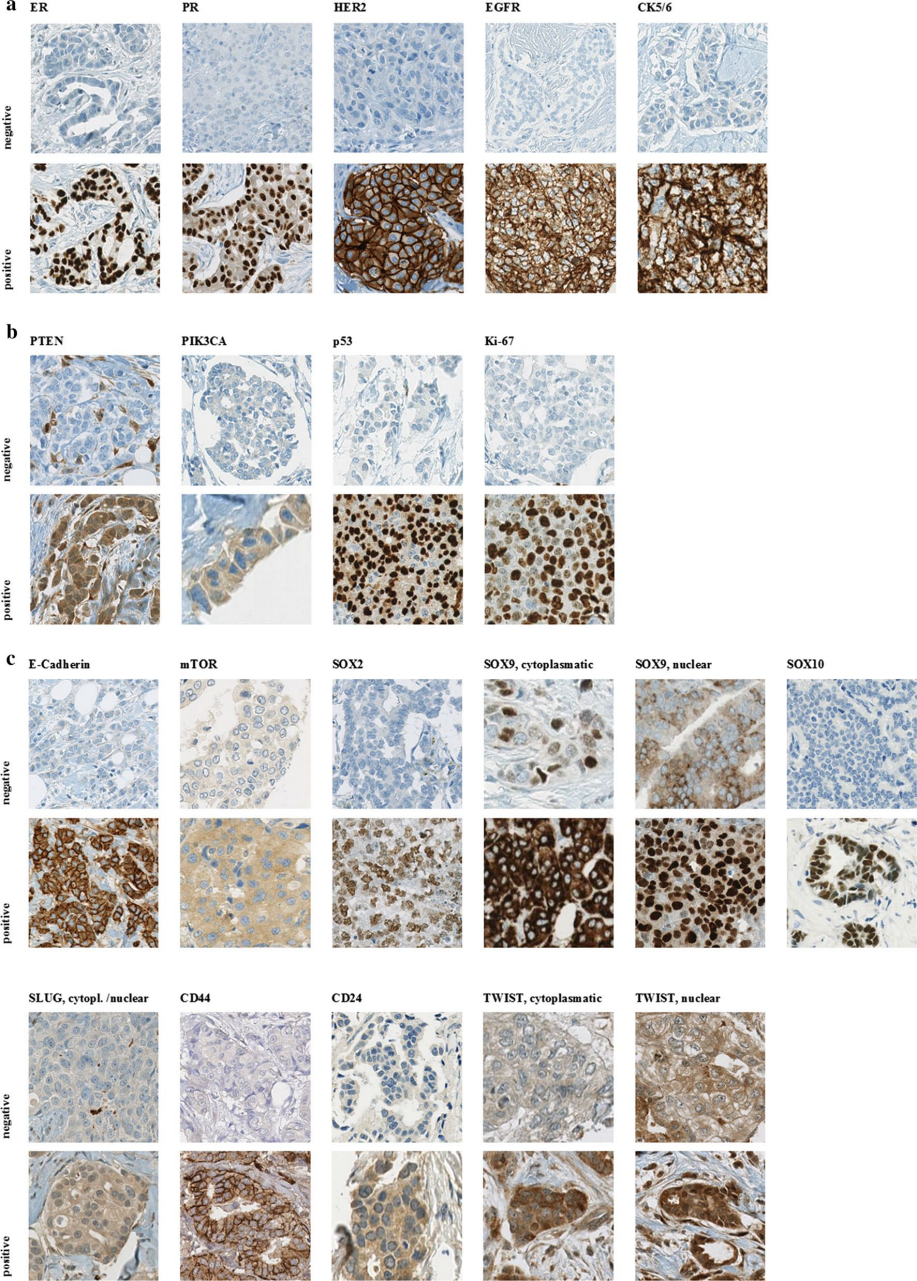
Representative immunohistochemical stains according to biological features. **a** Conventional predictive/prognostic markers, **b** tumor progression markers, **c** stem cell markers

Representative stains from each stain are shown in Fig. 1.

### Fluorescent in situ hybridization (FISH) reaction

#### HER2

The HER2 gene was tested by using a dual fluorescence kit (PathoVysion, Vysis, Abbott AG, Diagnostic Division Baar, Switzerland) containing the HER2 gene (17q11.2-q12, directly labeled with fluorescent spectrum orange) and CEP17 (17p11.1-q11.1, labeled with fluorescent spectrum green). The whole procedure was carried out using the PathoVysion probes on the fully automated Leica Bond autostainer (Leica Biosystems, Nunningen, Switzerland).

#### PTEN

The PTEN gene was visualized by the Vysis PTEN probe (Vysis, Abbott AG, Diagnostic Division Baar, Switzerland). The kit contained the PTEN gene (10q23) labelled with orange and the CEP10 probe (10p11.1-q11.1) labeled with green. The procedure was carried out using the Leica Bond autostainer according to the manufacturer’s instructions.

#### PIK3CA

The Vysis PIK3CA dual probe (labelled as green, locus 3q26.32) together with the CEP3 probe (labelled as orange, locus 3p11.1-q11.1) were used (Vysis, Abbott AG, Diagnostic Division Baar, Switzerland). The procedure was done on the Leica Bond autostainer.

### Scoring of fluorescent in situ hybridization (FISH) reactions

#### HER2

**Fig. 2:**
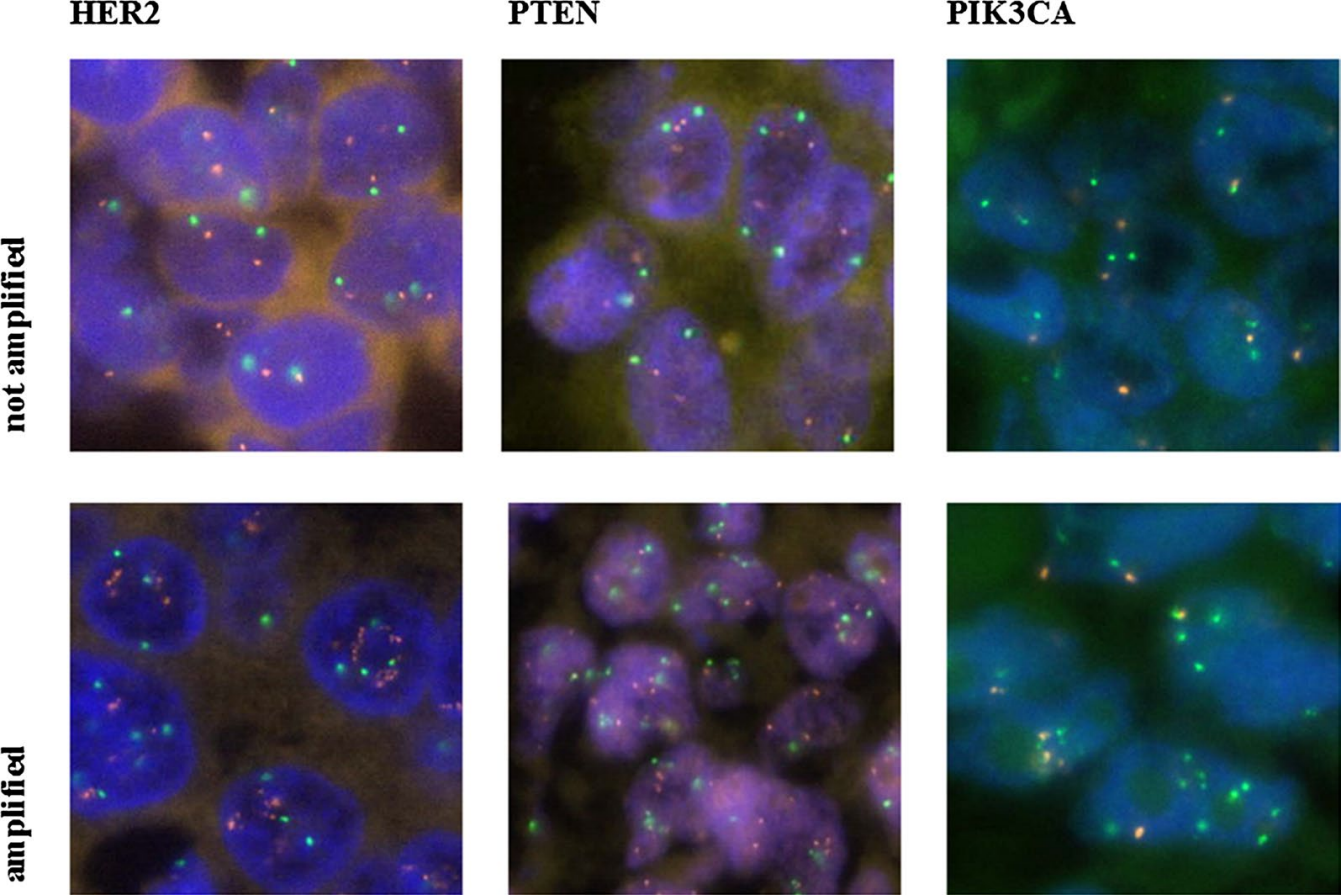
Representative areas of FISH reactions with negative and amplified samples: HER2, PTEN, PIK3CA

The 2013 ASCO-CAP guidelines were used for interpreting the signals in the FISH tests [16]. The number of signal copies and the ratios (*HER2/CEP17*) were calculated for each probe. Gene copies (> 6 in at least 10% of the tumor surface) or cluster formations (small clusters ~ 6 copies, larger clusters ~ 12 copies) were defined as amplified. Similarly, a ratio > 2 was defined as amplified (positive), a ratio < 2 was set as negative (Fig. 2).

#### PTEN, PIK3CA

Scoring was done in an analogous way as in HER2 FISH assays described above.

Illustrative areas of FISH reactions are shown in Fig. 2.

### Transcriptome (mRNA) analysis

15 cases, five from each histological grading (5 cases G1, 5 cases G2, 5 cases G3) were selected for transcriptome analysis using next generation sequencing. The same central and peripheral areas of the paraffin blocks that were used for TMA construction were punched for reception of material for RNA extraction. HE slide controls were used for the area selection for the mRNA tissue preparation.

Paraffin blocks were punched both in the center and in the tumor periphery. RNA was isolated from these punches using the Maxwell FFPE RNA isolation kit (Promega) according to the manufacturer’s manual in the Institute of Pathology and Molecular Pathology University Hospital Zürich. The RNA amount was quantified by Qubit measurement. The fragment size of the RNA was determined by the DV_200_ analysis using the bioanalyzer. Transcriptome analysis was performed at the Functional Genomics Center Zurich, Switzerland.

The SMARTer Stranded Total RNA-Seq Kit-Pico Input Mammalian (Clontech Laboratories, Inc., A Takara Bio Company, California, USA) was used in the preparation of libraries for sequencing. No fragmentation was performed. Total RNA samples (10 ng) were reverse-transcribed using random priming into double-stranded cDNA in the presence of a template switch oligo (TSO). When the reverse transcriptase reaches the 5′ end of the RNA fragment, the enzyme’s terminal transferase activity adds non-templated nucleotides to the 3′ end of the cDNA. The TSO pairs with the added non-templated nucleotide, enabling the reverse transcriptase to continue replicating to the end of the oligonucleotide. This resulted in a cDNA fragment that contains sequences derived from the random priming oligo and the TSO. PCR amplification using primers binding to these sequences was performed. The PCR added full-length Illumina adapters, including the index for multiplexing. Ribosomal cDNA was cleaved by ZapR in the presence of the mammalian-specific R-Probes. Remaining fragments were enriched with a second round of PCR amplification using primers designed to match Illumina adapters.

The quality and quantity of the enriched libraries were validated using the Tapestation (Agilent, Waldbronn, Germany). The product is a smear with an average fragment size of approximately 360 bp. The libraries were normalized to 10 nM in Tris–Cl 10 mM, pH8.5 with 0.1% Tween 20. The libraries were sequenced single end 125 bp using the Illumina HiSeq 4000 (Illumina, Inc, California, USA).

The RNA-seq data analysis consisted of the following steps:

Read-alignment was done with STAR (version 2.5.3a). As reference, we used the Ensembl genome build GRCh38.p10. With the gene annotations of Ensembl release 91.

The STAR alignment options were “–outFilterType BySJout–outFilterMatchNmin 30–outFilterMismatchNmax 10–outFilterMismatchNoverLmax 0.05–alignSJDBoverhangMin 1–alignSJoverhangMin 8–alignIntronMax 1,000,000–alignMatesGapMax 1,000,000–outFilterMultimapNmax 50”.

Gene expression values were computed with the function featureCounts from the R package Rsubread.

Differential expression was computed using the generalized linear model implemented in the Bioconductor package EdgeR. In the statistical model, we considered as factors the tumor area and the patient [17–19].

### Statistical analysis

IBM SPSS Statistics 23 was used for statistical analysis of the correlations of intratumoral heterogeneity and grading to stem cell characteristics and tumor progression markers. Frequency distribution was showed using crosstabs as a multivariate statistical method. Chi square and Spearman rank correlation coefficient were calculated to estimate the strength of the correlation as well as fisher’s exact test. p-value < 0.05 were interpreted as statistically significant. Correlation matrix graphics were constructed using the Correlation Matrix STHDA.com online software.

## Results

### Intra-tumoral heterogeneity

Expression levels of all markers were compared between tissue cores of central and peripheral tumor parts to assess intra-tumoral heterogeneity. Comparison with tissue samples of lymph node metastasis was limited due to small number of samples. All three groups of markers (1) conventional predictive markers (ER, PR, HER2, EGFR and CK5/6), (2) tumor progression markers (PTEN, PIK3CA, p53, Ki-67) and (3) stem cell markers (E-cadherin, mTOR, SOX2, SOX9, SOX10, SLUG, CD44, CD24 and TWIST) were analyzed. There was no significant difference in the expression level in any of the analyzed markers in terms of central and peripheral tumor areas. Central and peripheral spots showed a high correlation (as detailed in Additional file 2: Table S2). Additionally, there was a complete concordance between central tumor areas and metastatic lesions in all marker expression.

### Correlation to histological grading

All previously described markers were correlated to histopathological grading. Conventional parameters (ER, PR, HER2 status, EGFR, CK5/6), as expected significantly correlated to histological grading (p < 0.05).

Tumor progression markers as PIK3CA FISH amplification, p53 and Ki-67 immunohistochemical expression showed a strong significant correlation between histological grading and expression level (p < 0.0001). PTEN both with immunohistochemistry and FISH correlated slightly with histological grading (p < 0.05). PIK3CA protein expression did not show significant correlation to grading.

Among stem cell markers, a strong significant correlation between histological grading and expression level was found with E-cadherin, SOX2, SOX10, CD24 and TWIST. Furthermore, a slight significant correlation was seen with SOX9 (cytoplasmatic), SLUG (cytoplasmatic) and CD44. SOX9 (nuclear) and mTOR expression showed no correlation to histological grading.

**Fig. 3:**
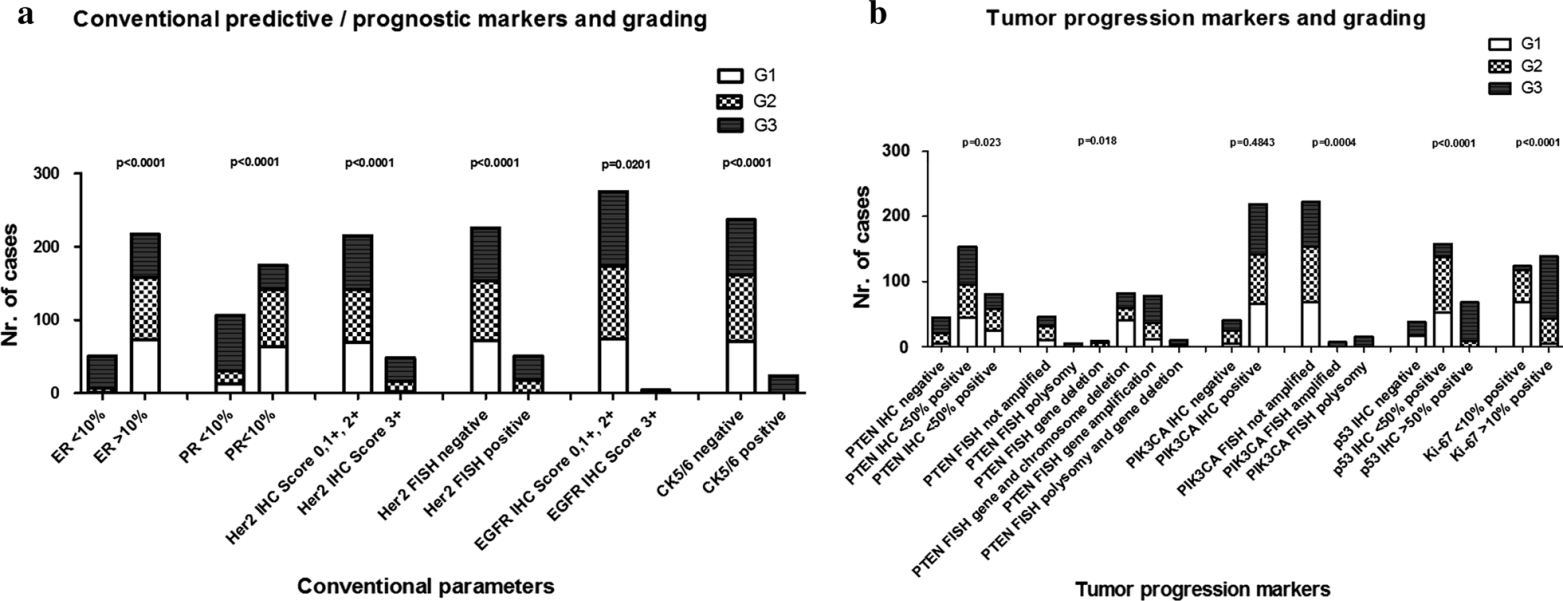
Graphical illustration of association between histological grading (**a**) and conventional prognostic/predictive markers (**b**) and tumor progression markers. p-values reflect Fisher’s exact test results

**Fig. 4:**
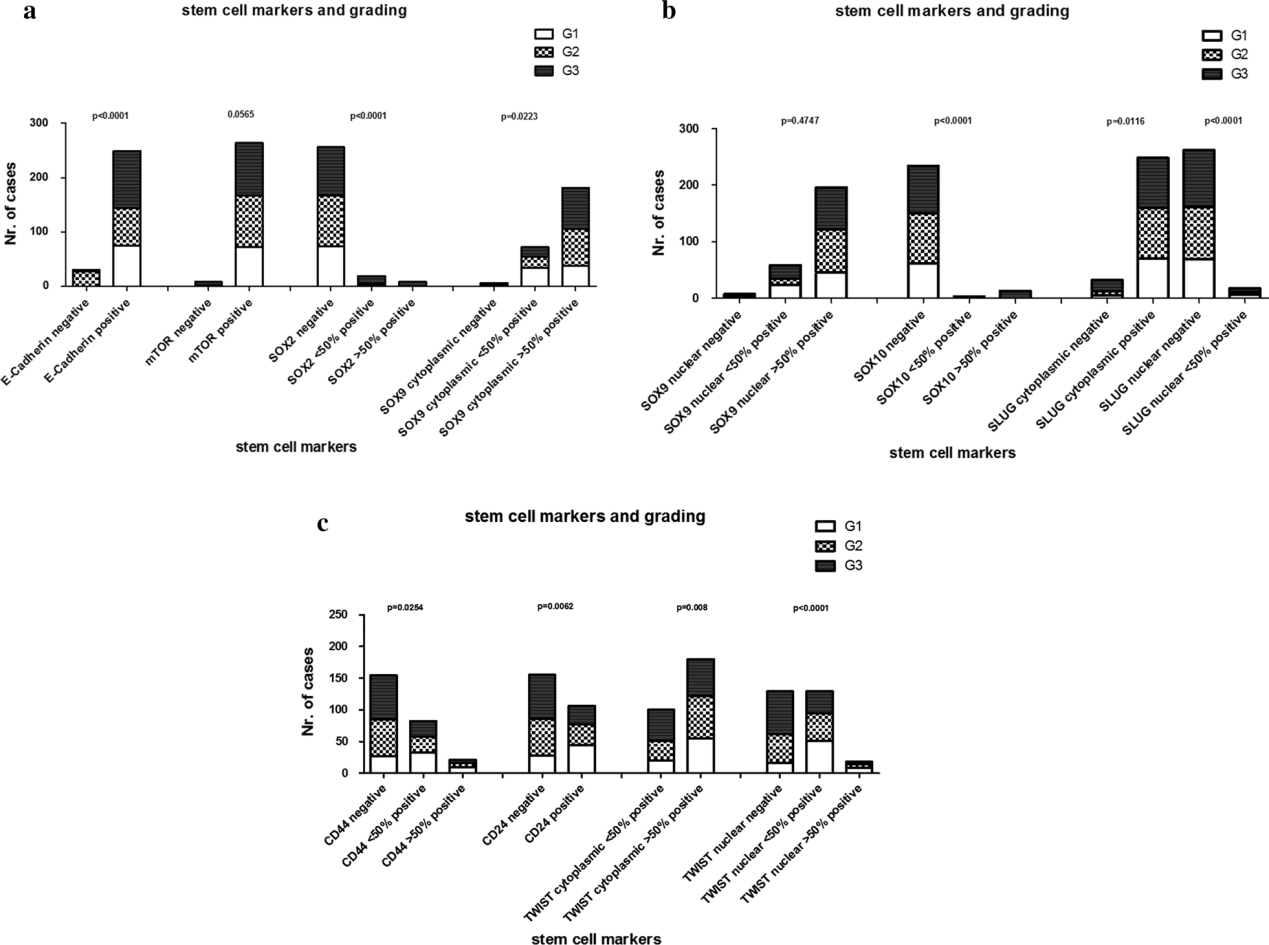
**a**–**c** Graphical illustration of association between histological grading and stem cell markers. p-values reflect Fisher’s exact test results

Details of the individual stains are shown in Additional file 3: Table S3 and in Figs. 3a, b, 4a–c.

### Correlation of conventional prognostic/predictive markers with stem cell and progression markers

Among the conventional predictive markers, a strong significant correlation was found between estrogen receptor and all the other conventional predictive markers, as well as between progesterone receptor and EGFR, HER2 IHC and CK5/6, between HER2 IHC and CK5/6 and HER2 FISH and between EGFR and CK5/6.

When correlated to tumor progression markers, a slight significant correlation was seen between estrogen receptor and PTEN (IHC and FISH), estrogen receptor and PIK3CA (IHC) as well as between progesterone receptor and PIK3CA (IHC). Comparison of ER and PR to Ki-67, HER2 IHC to PTEN FISH, p53 and Ki-67, as well as comparison of EGFR to p53 and Ki-67 and CK5/6 to PIK3CA, p53 and Ki-67 revealed a strong significant correlation.

The correlation of conventional predictive markers to stem cell markers showed numerous significant correlations. Especially the hormone receptors and EGFR revealed a strong significant correlation to stem cell markers.

**Fig. 5:**
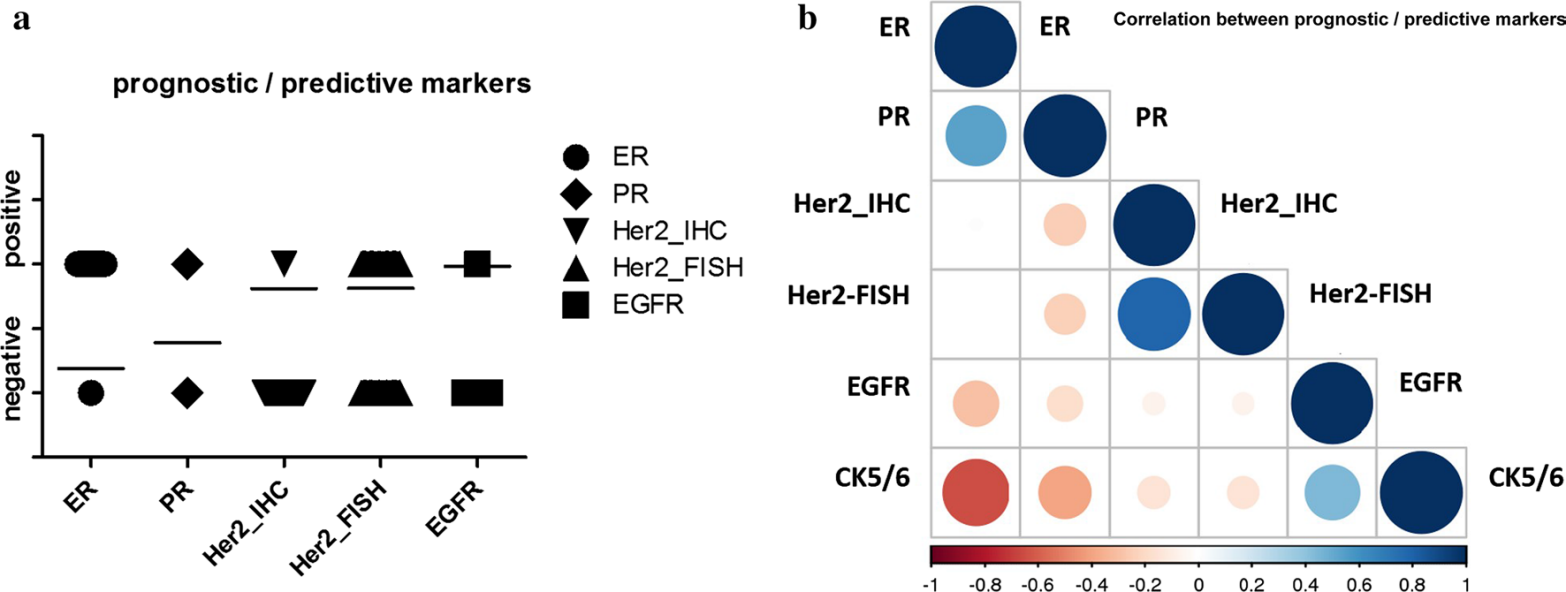
Graphical illustration of stain distribution (**a**) and correlation (**b**) among prognostic-predictive markers. Bars indicate mean values

Details of stain distribution and significant correlations are shown in Additional file 4: Table S4 and in Fig. 5a, b.

### Correlation of tumor progression markers to other markers

Correlation of the tumor progression markers PTEN, PIK3CA, p53 and Ki-67 to other tumor progression markers as well as to conventional predictive markers and stem cell markers was investigated.

Among the tumor progression markers, a significant correlation was only seen between PTEN and PIK3CA (IHC) and p53 and PIK3CA (IHC).

Tumor progression markers and stem cell markers revealed numerous strong significant correlations with each other. No significant correlations could be seen between tumor progression markers and SOX9 and CD44.

Comparison of tumor progression markers to conventional predictive markers is described above.

**Fig. 6:**
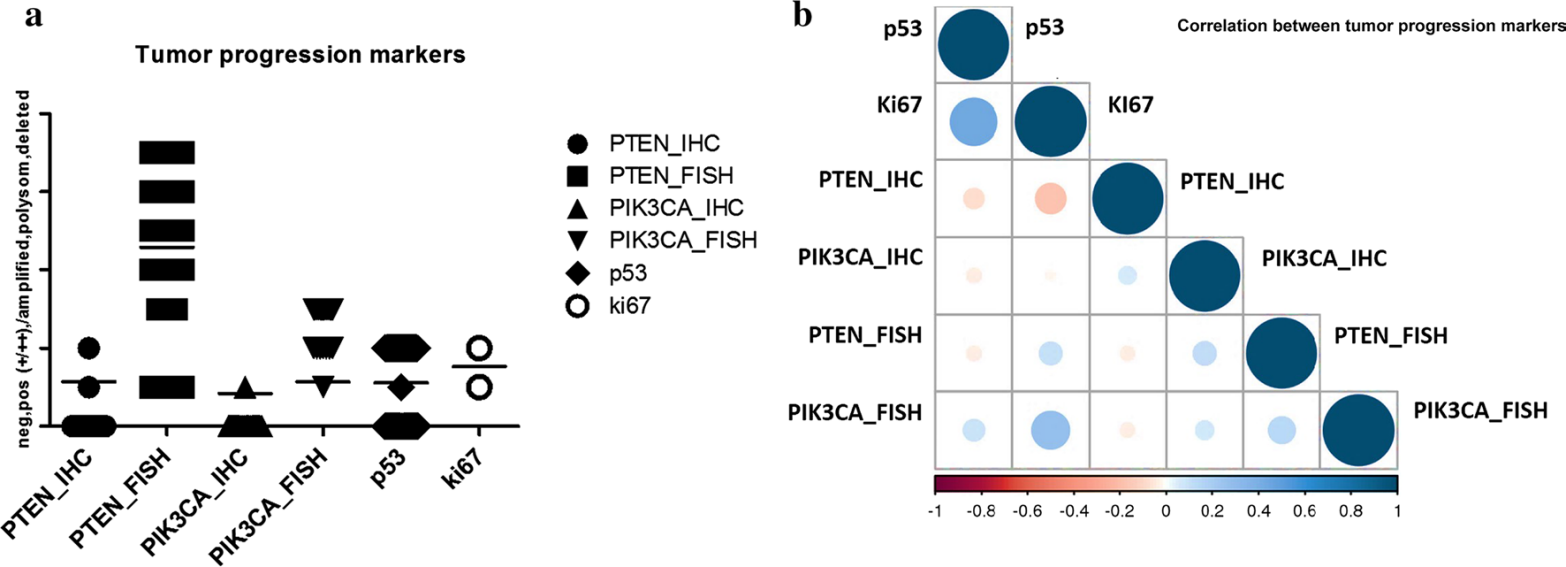
Graphical illustration of stain distribution (**a**) and correlation (**b**) among tumor progression markers. Bars indicate mean values

Details of stain distribution and significant correlations are shown in Additional file 5: Table S5 and in Fig. 6a, b.

### Comparison of stem cell markers to other markers

Comparison of stem cell markers to conventional markers and tumor progression markers is described above.

Among the stem cell markers numerous significant correlations were found. No significant correlation was seen between SOX10 and other stem cell markers.

**Fig. 7:**
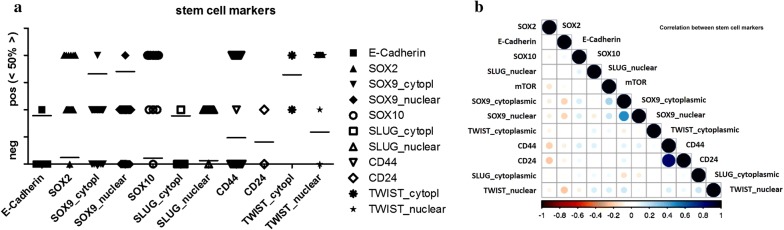
Graphical illustration of stain distribution (**a**) and correlation (**b**) among stem cell markers. Bars indicate mean values

Details of stain distribution and significant correlations are shown in Additional file 6: Table S6 and in Fig. 7a, b.

### Transcriptome (mRNA) analysis

**Fig. 8:**
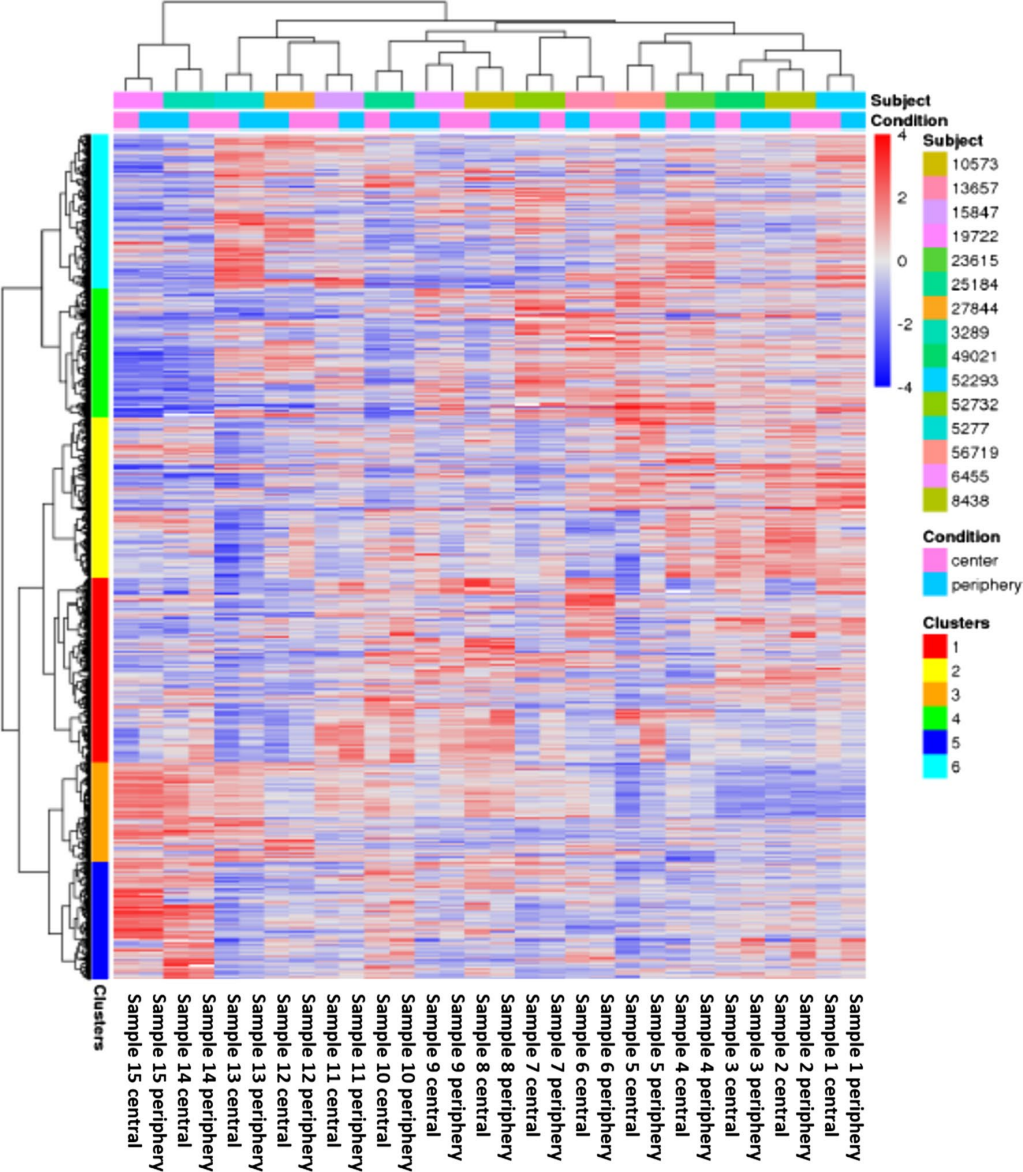
Heatmap showing the most variable 2000 genes in the RNA-Seq data. Sample and gene clustering was applied. The color indicates the log2-foldchange in comparison to the overall samples mean of the corresponding gene

**Fig. 9:**
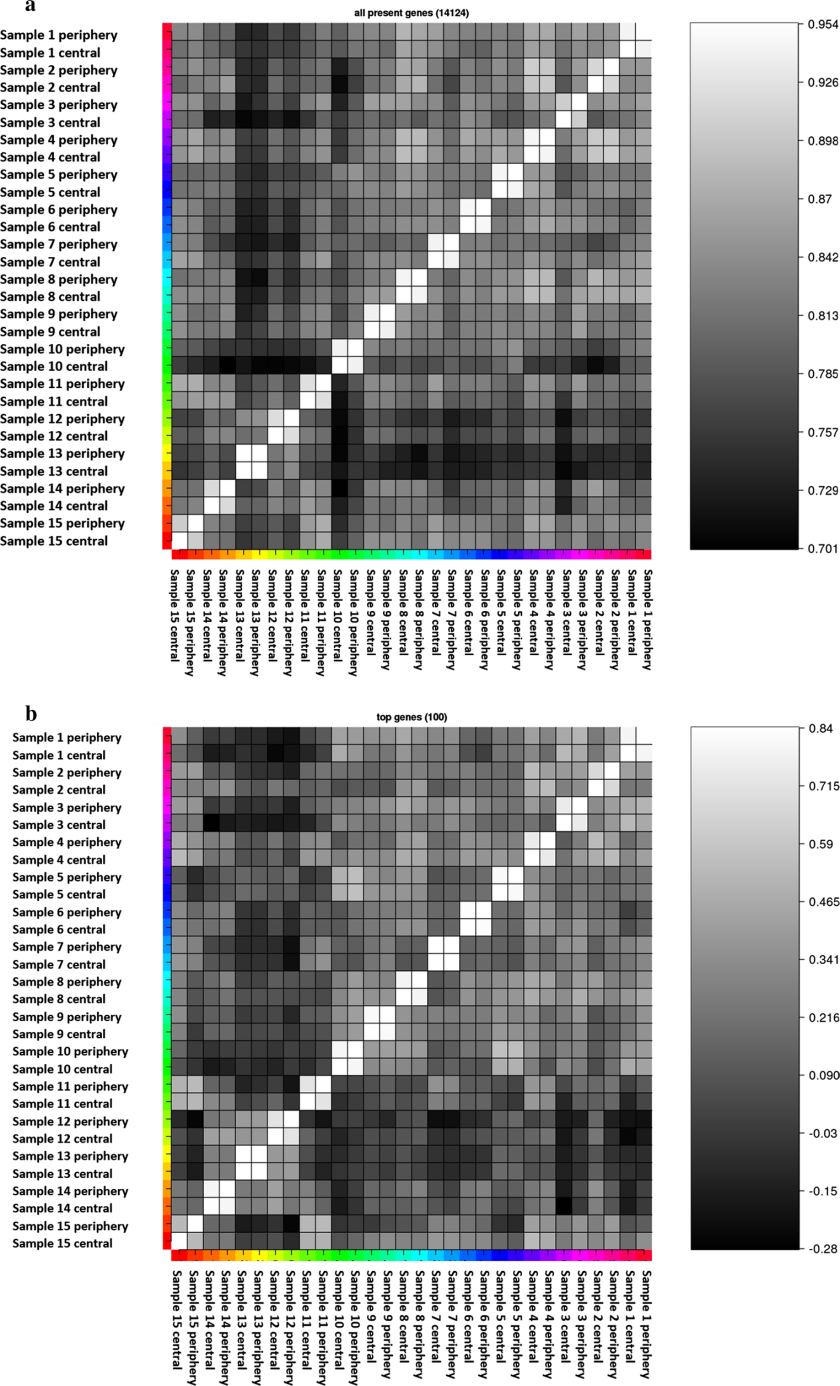
Heatmap showing the sample to sample correlation based on Kendall’s rank correlation coefficient for **a** all expressed genes (mean count > 20) and **b** 100 most variable genes across all samples

The sample clustering for the 2000 most variable genes in the experiment of the mRNA expression profiles shows that samples from the same patient are grouping together (Fig. 8). There is no indication for a differential expression signature between tumor areas based on the explorative cluster analysis. The sample to sample correlation illustrated in Fig. 9a confirms a strong positive correlation for all patient sample pairs included in this study. Focusing only on the 100 most variable genes provides very similar results (Fig. 9b).

**Fig. 10:**
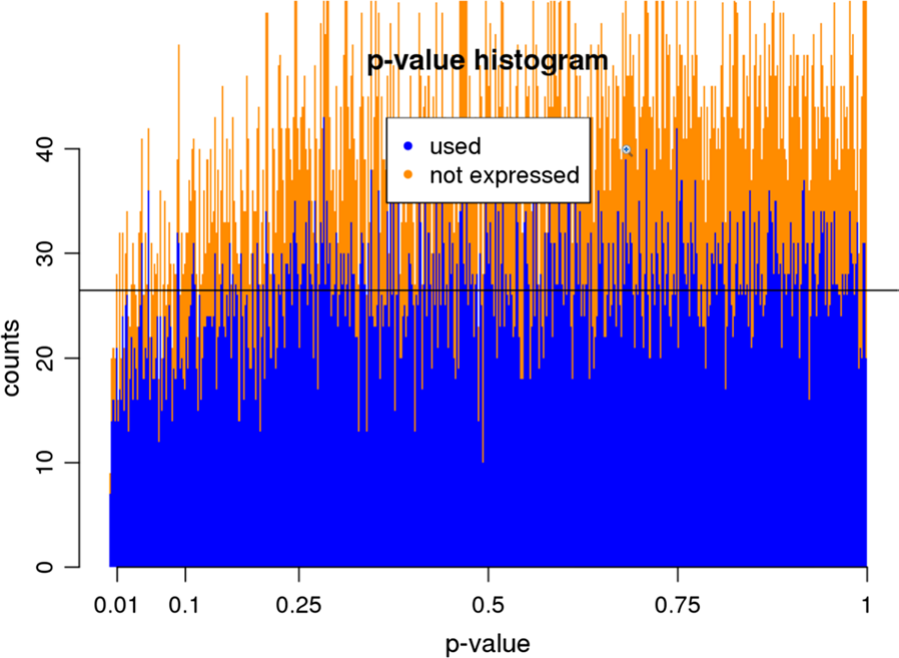
p-value histogram from the differential expression analysis. Absent and present genes in the data are separated by the color

Furthermore, the expression profiles between central and peripheral tumor areas were directly compared considering the patient as an additional factor in the linear model to remove the influence of different genetic backgrounds. The resulting *p* value histogram for all genes is following a uniform distribution (Fig. 10). This suggests that there is no gene signature which significantly differs between both tumor areas. No candidate gene was detected by applying a fold-change cut off > 2 and p-value ≤ 0.01.

Based on these results, there is no indication for any differentially expressed gene between periphery and center in this data.

## Discussion

In our study, we addressed the question whether a tissue micro array based approach can sufficiently detect intra-tumoral heterogeneity in stem cell and tumor progression markers in breast cancer by comparing expression levels between central and peripheral tumor parts. Our results suggest, that based on protein and DNA expression levels in tissue micro array core sections, no reliable distinction could be determined.

We are not aware of any similar approach in the literature describing the value of TMA in the intra-tumoral heterogeneity of stem cell and tumor progression markers in breast cancer.

Our findings partially contradict previous studies revealing intra-tumoral heterogeneity in breast cancer in tissue section based in the expression level of prognostic markers [9]. The finding of intra-tumoral heterogeneity in Ki-67 expression levels was confirmed in a recent study by Focke et al. [20]. On the other hand, when comparing tissue array-based measures of ER and PR status with immunohistochemistry, intra-tumoral heterogeneity, although present, did not affect exact tumor classification [21]. Similarly, in a study of Yang et al. investigating spatial heterogeneity of breast cancer stem cell markers, few patients revealed intra-tumoral heterogeneity, but no association with prognosis was seen [22].

Contrariwise, our results are in accordance to the data of Alkatout et al. where as well no difference of the expression levels of SLUG and TWIST between tumor center and tumor periphery was seen [23].

Spatial intra-tumoral heterogeneity has been described in connection with epithelial-mesenchymal transition (EMT), as breast cancer genome has been shown to change dynamically during cancer development from primary to metastatic tumor [24]. EMT is a process during which epithelial cells lose their epithelial cell characteristics and acquire mesenchymal phenotype and EMT cells gain motility and become invasive [25–29]. Hence, EMT has been considered as an important mechanism in tumor cell progression and metastasis, and consistently, the expression of EMT markers has been shown to be associated with poor prognosis [25–29]. There is increasing evidence that EMT is associated with acquisition of stem-cell properties [29, 30]. In a study of well-differentiated colorectal cancers and their metastasis a dedifferentiation and loss of epithelial properties was seen at the invasive tumor margin, and Brabletz et al. therefore proposed occurrence of EMT preferably at peripheral tumor sites [23, 31]. However, this hypothesis could not be confirmed by Alkatout et al. No significant difference of expression levels of the EMT markers SLUG, TWIST, SNAIL and Zeb1 between tumor center and tumor periphery could be revealed [23]. On the other hand, Connor et al. found a higher proliferative activity (Ki-67 index) at the tumor periphery than in the tumor center [32]. As described above, our results are in accordance to the data of Alkatout et al. We could not find a difference of expression levels of SLUG and TWIST between tumor center and periphery either. A possible interpretation of these findings is that epithelial-mesenchymal transition is not only occurring at the tumor periphery, but at multiple sites within the tumor [23].

The second aim of the study was to proof prognostic value of the special TMA containing different tumor areas by comparing expression levels of conventional predictive tumor markers, tumor progression markers and stem cell markers with histopathological grading. In breast cancer diagnostics hormone receptors (ER, PR) as well as HER2 are established prognostic and predictive biomarkers [33–35]. Based on the current TMA, we could proof a strong negative correlation between hormone receptor expression levels and histopathological grading and a significant positive correlation between histopathological grading and HER2/EGFR/CK5/6 expression, corresponding with literature data and confirming prognostic value of the TMA independently of the site of the biopsy was taken.

Ki-67 and p53 are connected to cell proliferation and if increased or overexpressed are associated with higher grading, metastatic potential and shortened overall-survival [33, 35–37]. A shortened time to progression and decreased survival has furthermore been seen with PTEN deletion and PIK3CA activating mutations [38, 39]. In accordance with these findings we found a significant positive correlation between Ki-67, p53 and PIK3CA expression levels and histopathological grading, whereas a negative correlation was seen with PTEN expression.

Previous studies about the predictive value of CD44 and CD24 have showed controversial results. In 2003, a CD44+/CD24− phenotype was first identified as a highly tumorigenic subpopulation of tumor cells with stem-cell-like characteristics [5, 6, 8]. Tumors showing this phenotype were described to favor occurrence of distant metastasis, but no significant correlation with clinical outcome was seen [40–43]. In studies separately analyzing CD44 as a predictive marker an association between CD44 positivity and increased progression-free and disease-free survival was found [44, 45]. The role of CD24 as a predictive marker was as well discussed controversial. The data of Kristiansen et al. revealed an association to shortened disease-free-survival, while Jang et al. found no significant correlation to prognosis [46, 47]. In our study, a slight respectively strong negative correlation between CD44 and CD24 and histopathological grading was seen, being concordant with the results of Dan et al. and Diaz et al.

TWIST, SLUG and SOX9 have lately been described as cancer stems cell markers inducing EMT [48–51]. In consistence with our findings, previous studies found these markers to be associated with higher histopathological grading and shortened overall survival [48, 50–55].

Through loss of E-cadherin tumor cells gain the ability to migrate and E-cadherin negativity was found to correlate with higher tumor grade and stage and poor prognosis [56, 57]. However, in contrast to these results, our data revealed a positive correlation to histopathological grading.

SOX2 and SOX10 are often present in breast cancer, although their role is poorly understood yet. Our data further support recent suggestions that SOX2 and SOX10 correlate to higher histopathological grading and hence to reduced overall survival [51, 58].

mTOR is part of a pathway playing an important role in tumorigenesis. Our findings are in agreement with a recent study of Ding et al. where no significant correlation between mTOR expression and clinical outcome was seen [59].

Intratumoral heterogeneity of tumor infiltrating lymphocytes (TIL) was addressed in a previous study assessing different anatomical regions of breast cancer on large tissue sections and showing that TIL-s at the infiltrating margins are more numerous than in the intratumoral stromal tissue [60]. Interestingly, the same trend was found when metastatic lesions were compared with the primary tumor suggesting that the primary tumor most likely determines the TIL composition in the metastatic lesion which mirrors the immunological pattern of primary tumor [60].

Regarding the correlation between stem cell, tumor progression and conventional prognostic markers, we could show in our study, that within the current TMA cohort most markers displayed significant inter-marker correlation, independently from the anatomical site of the tumor sample taken. Most breast tumors are estrogen receptor and progesterone receptor positive and are associated with better overall survival. Further, HER2 negativity was found to correlate with ER and PR positivity and was as well described to be associated with favorably prognosis [33, 35, 38]. Ki-67, as a proliferation marker, as well as p53 are linked with poor prognosis. Their expression levels negatively correlate with ER and PR expression levels and positively correlate with HER2 status [33, 37, 61, 62]. All these findings are in complete agreement with the results of our study, where a strong significant correlation between the hormone receptors and a negative correlation between ER/PR and HER2, Ki-67 and p53 was seen. On the other hand, no correlation was found between p53 and Ki-67 expression levels.

Trastuzumab is a monoclonal antibody, used for treatment of metastatic HER2-positive breast cancer. Lately, it was suggested that mutations of the PI3 K/AKT pathway and loss of PTEN could interfere with trastuzumab treatment and lead to resistance. Recent studies therefore questioned whether PIK3CA and PTEN could be used as predictive markers of trastuzumab efficacy [38, 63]. We revealed a strong significant correlation between HER2 status and PTEN expression level and between PTEN and PIK3CA but not between HER2 status and PIK3CA expression level was seen. The results agreed with previous studies of Razis et al. and Lebok et al., where as well a correlation between HER2 and PTEN was found [38, 39]. So far, PTEN loss and PIK3CA mutations are proposed to be mutually exclusive, what seems to be inconsistent with our finding of a significant correlation between the two markers, even though mutation status of PIK3CA was not assessed in our study [38]. However in one recent study by Sueta et al., a combined analysis of PTEN loss and PIK3CA mutations was shown to potentially identify patients who are unlikely to respond to trastuzumab therapy in neoadjuvant setting [64]. It seems, that complete pathological response, pCR is lower if an activating PIKC3CA mutation and/or low level or PTEN expression is present [64]. PTEN is a negative regulator of the PIK3CA pathway, consequently, simultaneous PIK3CA mutations and low PTEN expression can stronger predict response to trastuzumab therapy then one marker alone, which can potentially identify patients who can benefit from PIK3 K targeted therapies [64]. The negative and positive correlation between PIKC3CA and PTEN protein expression and gene copy numbers in our study corroborate with these observations.

So far, only few studies investigated the association of CD24/CD44 to other markers. Collina et al. described a slight statistical link between CD44 and Ki-67, which could not be confirmed by our study [43]. Jang et al. previously found a negative, respectively positive correlation between CD44/CD24 and HER2 status [47]. Our data revealed as well an inverse correlation between HER2 and CD44, but inconsistent with the results of Jang et al. an inverse association to CD24 too.

The over-expression of most stem cell markers has been linked to poor prognosis and to hormone receptor negativity and HER2 positivity, which also could be confirmed in our data [48, 54, 56, 58, 65]. The numerous correlations among the stem cell markers, further providing a similar predictive value [48, 54, 56, 58, 65].

## Conclusion

Based on the data in our study, a tissue micro array based approach failed to deliver sufficient information on intra-tumoral heterogeneity in breast cancer stem cell and tumor progression markers by comparing central and peripheral tumor areas. Further studies using single cell genomic alterations including further mRNA based assessments are needed to fully understand the role and interaction between stem cell marker and intra-tumoral heterogeneity also in terms of tumor progression in breast cancer. On the other hand, the anatomical site of the tumor samples did not influence prognostic information on these markers which was independent from the site of biopsy was taken.

## Additional files

**Additional file 1: Table S1.** Immunohistochemical stains and laboratory details.

**Additional file 2: Table S2.** Analysis of intratumoral heterogeneity.

**Additional file 3: Table S3.** Correlation to histopathological grading.

**Additional file 4: Table S4.** Conventional predictive/prognostic markers—significant correlations.

**Additional file 5: Table S5.** Tumor progression markers—significant correlations.

**Additional file 6: Table S6.** Stem cell markers—significant correlations.
