# Correction to: Limited utility of tissue micro-arrays in detecting intra-tumoral heterogeneity in stem cell characteristics and tumor progression markers in breast cancer

**DOI:** 10.1186/s12967-018-1553-0

**Published:** 2018-07-02

**Authors:** Pascale Kündig, Charlotte Giesen, Hartland Jackson, Bernd Bodenmiller, Bärbel Papassotirolopus, Sandra Nicole Freiberger, Catharine Aquino, Lennart Opitz, Zsuzsanna Varga

**Affiliations:** 1 Institute of Pathology and Molecular Pathology, University Hospital Zurich, Schmelzbergstrasse 12, 8091 Zurich, Switzerland; 2 Institute of Molecular Life Sciences, University of Zurich, Zurich, Switzerland; 3 Breast Center Seefeld, Zurich, Switzerland; 4 Functional Genomics Center Zurich, Zurich, Switzerland

## Correction to: J Transl Med (2018) 16:118 10.1186/s12967-018-1495-6

Following publication of the original article [1], a typesetting mistake is reported. For Fig. 7b, a copy of Fig. 6b has been published. The correct Fig. 7b is given in this correction and the original article has been updated.

**Fig. 7:**
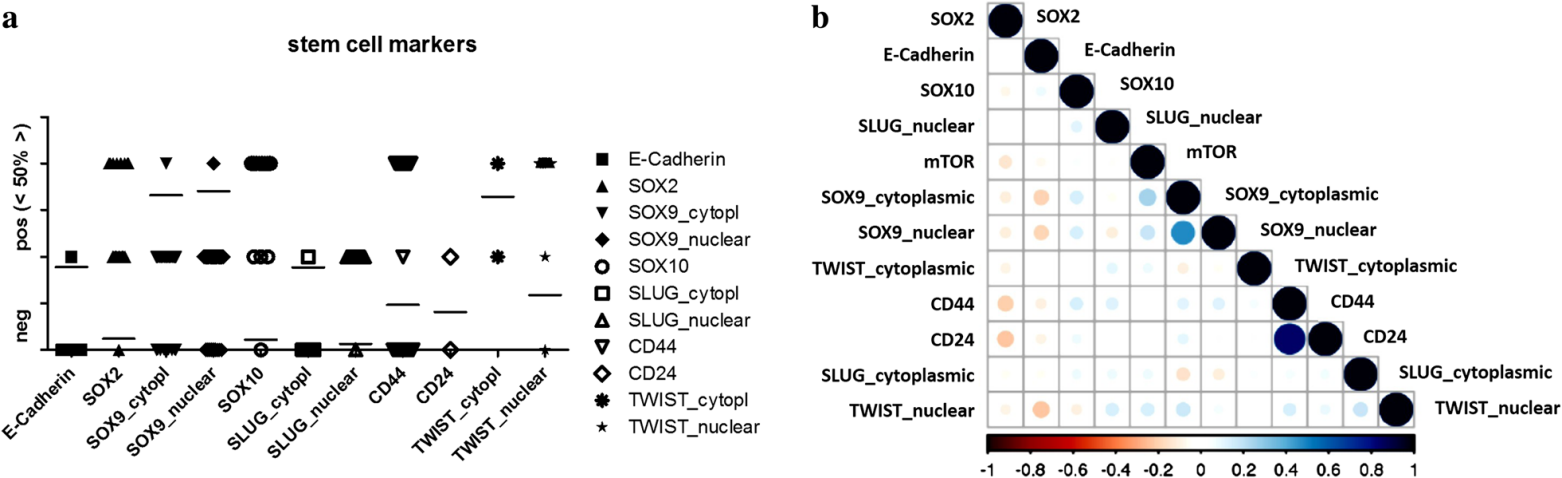
Graphical illustration of stain distribution (**a**) and correlation (**b**) among stem cell markers. Bars indicate mean values

The publisher apologizes to the authors and readers for the inconvenience.
