# Supplementary material for: Limited utility of tissue micro-arrays in detecting intra-tumoral heterogeneity in stem cell characteristics and tumor progression markers in breast cancer
Source: J Transl Med. 2018 May 8;16:118. doi: 10.1186/s12967-018-1495-6 (PMC5941467; doi:10.1186/s12967-018-1495-6)
Supplement: Supplementary file 1 — Additional file 1: Table S1. Immunohistochemical stains and laboratory details. [file 12967_2018_1495_MOESM1_ESM.docx]

**Additional file 1: Table S1. Immunohistochemical stains and laboratory details**

|  | **clone** | **dilution** | **pretreatment** | **visualisation** | **staining machine** |
| --- | --- | --- | --- | --- | --- |
| **Estrogen receptor** | SP1 | prediluted | CC1 m | UltraView | Ventana Autostainer |
| **Progesterone receptor** | 1E2 | prediluted | CC1 52 | UltraView | Ventana Autostainer |
| **HER2** | 4B5 | prediluted | CC1 m | UltraView | Ventana Autostainer |
| **EGFR** | 31G7 | 1:50 | P1 | OptiView | Ventana Autostainer |
| **E-Cadherin** | EP700Y | 1:200 | CC1 40 | OptiView | Ventana Autostainer |
| **CK 5/6** | D5 | prediluted | CC1 48 | OptiView | Ventana Autostainer |
| **p63** | 4A4 | prediluted | CC1 64 | OptiView | Ventana Autostainer |
| **PTEN** | 6H2.1 | 1:100 | Bond H2 | Refine HRP | Leica Bond Autostainer |
| **PIK3CA** | Sigma HPA009985 polyclonal | 1:25 | CC1 standard | UltraMap Rabbit | Ventana Autostainer |
| **p53** | DO7 | 1:80 | CC1 32 | OptiView | Ventana Autostainer |
| **Mib1/Ki-67** | 30-9 | prediluted | CC1 32 | UltraMap Rabbit | Ventana Autostainer |
| **mTOR** | 49F9 | 1:100 | CC1 92 | OptiView | Ventana Autostainer |
| **SOX2** | EPR3131 | 1:100 | CC1 92 | OptiView | Ventana Autostainer |
| **SOX9** | AB5535 polyclonal | 1:400 | CC1 48 | OptiView | Ventana Autostainer |
| **SOX10** | BC34 | 1:150 | Bond H2 | Refine HRP | Leica Bond Autostainer |
| **SLUG** | C19G7 | 1:25 | CC1 standard | UltraMap Rabbit | Ventana Autostainer |
| **CD44** | G44-26 | 1:100 | CC1 8 | OptiView | Ventana Autostainer |
| **CD24** | SN3b | 1:300 | Bond H2 | Refine HRP | Leica Bond Autostainer |
| **TWIST** | Abcam ab 56581 | 1:200 | CC1 mild | UltraMap Rabbit | Ventana Autostainer |
