# Supplementary material for: Limited utility of tissue micro-arrays in detecting intra-tumoral heterogeneity in stem cell characteristics and tumor progression markers in breast cancer
Source: J Transl Med. 2018 May 8;16:118. doi: 10.1186/s12967-018-1495-6 (PMC5941467; doi:10.1186/s12967-018-1495-6)
Supplement: Supplementary file 2 — Additional file 2: Table S2. Analysis of intratumoral heterogeneity. [file 12967_2018_1495_MOESM2_ESM.docx]

**Additional file 2: Table S2. Analysis of intratumoral heterogeneity**

|  | **Pearson Correlation** | **Kendall's tau b** | **Spearman's rho** |
| --- | --- | --- | --- |
| ER | 0.998* | 0.978* | 0.985* |
| PR | 0.986* | 0.954* | 0.982* |
| HER2 (IHC) | 0.950* | 0.929* | 0.954* |
| HER2 (FISH) | 0.981* | 0.936* | 0.972* |
| EGFR | 1.000* | 1.000* | 1.000* |
| E-Cadherin | 0.800* | 0.800* | 0.800* |
| CK 5/6 | 1.000* | 1.000* | 1.000* |
| PTEN (IHC) | 0.838* | 0.821* | 0.847* |
| PTEN (FISH) | 0.804* | 0.849* | 0.900* |
| PIK3CA (IHC) | 0.969* | 0.969* | 0.969* |
| PIK3CA (FISH) | 0.804* | 0.747* | 0.762* |
| p53 | 0.874* | 0.865* | 0.889* |
| Ki-67 | 0.981* | 0.936* | 0.968* |
| mTOR | 0.369* | 0.369* | 0.369* |
| SOX2 | 0.957* | 0.923* | 0.923* |
| SOX9, cytoplasmatic | 0.916* | 0.941* | 0.959* |
| SOX9, nuclear | 0.943* | 0.949* | 0.956* |
| SOX10 | 0.968* | 0.896* | 0.902* |
| SLUG, cytoplasmatic | 0.887* | 0.887* | 0.887* |
| SLUG, nuclear | 0.884* | 0.884* | 0.884* |
| CD44 | 0.932* | 0.814* | 0.926* |
| CD24 | 0.984* | 0.984* | 0.984* |
| TWIST, cytoplasmatic | 0.845* | 0.845* | 0.845* |
| TWIST, nuclear | 0.896* | 0.874* | 0.880* |
| *. Correlation is significant at the 0.01 level (2-tailed). | | | |
