# Supplementary material for: Limited utility of tissue micro-arrays in detecting intra-tumoral heterogeneity in stem cell characteristics and tumor progression markers in breast cancer
Source: J Transl Med. 2018 May 8;16:118. doi: 10.1186/s12967-018-1495-6 (PMC5941467; doi:10.1186/s12967-018-1495-6)
Supplement: Supplementary file 3 — Additional file 3: Table S3. Correlation to histopathological grading. [file 12967_2018_1495_MOESM3_ESM.docx]

**Additional file 3: Table S3. Correlation to histopathological grading**

| **a) Conventional predictive/ prognostic markers** | | | | | | | | | | | |  |
| --- | --- | --- | --- | --- | --- | --- | --- | --- | --- | --- | --- | --- |
|  | | | | | | | | | | | |  |
|  | Grading | | | | | | | | Pearson Chi-Square (p-value) | Spearman Correlation (p-value) | Kappa (p-value) | Fisher's exact test (p-value) |
|  | G1 | | G2 | | G3 | | Total | |  |  |  |  |
|  | n | % | n | % | n | % | n | % |  |  |  |  |
| **ER** |  |  |  |  |  |  |  |  |  |  |  |  |
| ≤10% positive | 1 | 1% | 6 | 7% | 44 | 43% | 51 | 19% | 0.000 | 0.000 | 0.725 | <0.0001 |
| >10% positive | 73 | 99% | 85 | 93% | 59 | 57% | 217 | 81% |  |  |  |  |
| Total | 74 | 100% | 91 | 100% | 103 | 100% | 268 | 100% |  |  |  |  |
| **PR** |  | | | | | | | | | | | |
| ≤10% positive | 13 | 17% | 18 | 19% | 75 | 69% | 106 | 38% | 0.000 | 0.000 | 0.733 | <0.0001 |
| >10% positive | 64 | 83% | 78 | 81% | 33 | 31% | 175 | 62% |  |  |  |  |
| Total | 77 | 100% | 96 | 100% | 108 | 100% | 281 | 100% |  |  |  |  |
| **HER2 IHC** | | | | | | | | | | | | |
| negative/ weak positivity (0,1+,2+) | 70 | 97% | 72 | 84% | 73 | 70% | 215 | 82% | 0.000 | 0.000 | 0.051 | <0.0001 |
| strong positivity (3+) | 2 | 3% | 14 | 16% | 32 | 30% | 48 | 18% |  |  |  |  |
| Total | 72 | 100% | 86 | 100% | 105 | 100% | 263 | 100% |  |  |  |  |
| **HER2 FISH** |  | | | | | | | | | | | |
| not amplified | 72 | 100% | 82 | 83% | 72 | 68% | 226 | 82% | 0.000 | 0.000 | 0.017 | <0.0001 |
| amplified | 0 | 0% | 17 | 17% | 34 | 32% | 51 | 18% |  |  |  |  |
| Total | 72 | 100% | 99 | 100% | 106 | 100% | 277 | 100% |  |  |  |  |
| **EGFR** |  | | | | | | | | | | | |
| negative/ weak positivity (0,1+,2+) | 74 | 100% | 99 | 100% | 102 | 96% | 275 | 99% | 0.036 | 0.021 | 0.818 | 0.0201 |
| strong positivity  (3+) | 0 | 0% | 0 | 0% | 4 | 4% | 4 | 1% |  |  |  |  |
| Total | 74 | 100% | 99 | 100% | 106 | 100% | 279 | 100% |  |  |  |  |
| **CK 5/6** |  | | | | | | | | | | | |
| negative | 71 | 100% | 91 | 100% | 75 | 76% | 237 | 91% | 0.000 | 0.000 | 0.002 | <0.0001 |
| positive | 0 | 0% | 0 | 0% | 24 | 24% | 24 | 9% |  |  |  |  |
| Total | 71 | 100% | 91 | 100% | 99 | 100% | 261 | 100% |  |  |  |  |
|  |  |  |  |  |  |  |  |  |  |  |  |  |
| **b) Tumor progression markers** | | | | | | | | | | | |  |
|  | Grading | | | | | | | | Pearson Chi-Square (p-value) | Spearman Correlation (p-value) | Kappa (p-value) | Fisher's exact test (p-value) |
|  | G1 | | G2 | | G3 | | Total | |  |  |  |  |
|  | n | % | n | % | n | % | n | % |  |  |  |  |
| **PTEN IHC** |  |  |  |  |  |  |  |  |  |  |  |  |
| negative | 5 | 7% | 16 | 16% | 24 | 23% | 45 | 16% | 0.029 | 0.005 | 0.172 | 0.023 |
| ≤50% positive | 45 | 60% | 50 | 51% | 59 | 56% | 154 | 55% |  |  |  |  |
| >50% positive | 25 | 33% | 33 | 33% | 23 | 22% | 81 | 29% |  |  |  |  |
| Total | 75 | 100% | 99 | 100% | 106 | 100% | 280 | 100% |  |  |  |  |
| **PTEN FISH** |  | | | | | | | | | | | |
| not amplified | 10 | 16% | 21 | 29% | 15 | 16% | 46 | 20% | 0.000 | 0.010 | 0.904 | 0.0018 |
| polysomy | 0 | 0% | 0 | 0% | 5 | 5% | 5 | 2% |  |  |  |  |
| gene deletion | 0 | 0% | 6 | 8% | 3 | 3% | 9 | 4% |  |  |  |  |
| gene and chromosome deletion | 41 | 65% | 18 | 25% | 23 | 24% | 82 | 36% |  |  |  |  |
| heterogeneous gene amplification | 12 | 19% | 25 | 34% | 41 | 44% | 78 | 34% |  |  |  |  |
| polysomy and gene deletion | 0 | 0% | 3 | 4% | 7 | 7% | 10 | 4% |  |  |  |  |
| Total | 63 | 100% | 73 | 100% | 94 | 100% | 230 | 100% |  |  |  |  |
| **PIK3CA IHC** |  | | | | | | | | | | | |
| negative | 4 | 6% | 20 | 21% | 17 | 18% | 41 | 16% | 0.022 | 0.063 | 0.007 | 0.4843 |
| positive | 66 | 94% | 75 | 79% | 78 | 82% | 219 | 84% |  |  |  |  |
| Total | 70 | 100% | 95 | 100% | 95 | 100% | 260 | 100% |  |  |  |  |
| **PIK3CA FISH** |  |  | | | | | | | | | | |
| not amplified | 68 | 100% | 86 | 100% | 69 | 76% | 223 | 91% | 0.000 | 0.000 | 0.000 | 0.0004 |
| amplified | 0 | 0% | 0 | 0% | 7 | 8% | 7 | 3% |  |  |  |  |
| polysomy | 0 | 0% | 0 | 0% | 15 | 16% | 15 | 6% |  |  |  |  |
| Total | 68 | 100% | 86 | 100% | 91 | 100% | 245 | 100% |  |  |  |  |
| **p53** |  | | | | | | | | | | | |
| negative | 17 | 24% | 1 | 1% | 20 | 20% | 38 | 14% | 0.000 | 0.000 | 0.465 | <0.0001 |
| ≤50% positive | 53 | 76% | 84 | 89% | 21 | 21% | 158 | 60% |  |  |  |  |
| >50% positive | 0 | 0% | 9 | 10% | 59 | 59% | 68 | 26% |  |  |  |  |
| Total | 70 | 100% | 94 | 100% | 100 | 100% | 264 | 100% |  |  |  |  |
| **Ki-67** |  | | | | | | | | | | | |
| ≤10% positive | 68 | 94% | 49 | 56% | 7 | 7% | 124 | 47% | 0.000 | 0.000 | 0.000 | <0.0001 |
| >10% positive | 4 | 6% | 39 | 44% | 96 | 93% | 139 | 53% |  |  |  |  |
| Total | 72 | 100% | 88 | 100% | 103 | 100% | 263 | 100% |  |  |  |  |
|  |  |  |  |  |  |  |  |  |  |  |  |  |
| **c) Stem cell markers** | | | | | | | | | | | |  |
|  | Grading | | | | | | | | Pearson Chi-Square (p-value) | Spearman Correlation (p-value) | Kappa (p-value) | Fisher's exact test (p-value) |
|  | G1 | | G2 | | G3 | | Total | |  |  |  |  |
|  | n | % | n | % | n | % | n | % |  |  |  |  |
| **E-Cadherin** |  | | | | | | | | | | | |
| negative | 0 | 0% | 28 | 29% | 2 | 2% | 30 | 11% | 0.000 | 0.000 | 0.000 | <0.0001 |
| positive | 75 | 100% | 69 | 71% | 105 | 98% | 249 | 89% |  |  |  |  |
| Total | 75 | 100% | 97 | 100% | 107 | 100% | 279 | 100% |  |  |  |  |
| **mTOR** | | | | | | | | | | | | |
| negative | 0 | 0% | 2 | 2% | 6 | 6% | 8 | 3% | 0.066 | 0.020 | 0.085 | 0.0565 |
| positive | 72 | 100% | 95 | 98% | 97 | 94% | 264 | 97% |  |  |  |  |
| Total | 72 | 100% | 97 | 100% | 103 | 100% | 272 | 100% |  |  |  |  |
| **SOX2** |  | | | | | | | | | | | |
| negative | 73 | 97% | 94 | 96% | 89 | 81% | 256 | 90% | 0.000 | 0.000 | 0.012 | <0.0001 |
| ≤50% positive | 2 | 3% | 4 | 4% | 13 | 12% | 19 | 7% |  |  |  |  |
| >50% positive | 0 | 0% | 0 | 0% | 8 | 7% | 8 | 3% |  |  |  |  |
| Total | 75 | 100% | 98 | 100% | 110 | 100% | 283 | 100% |  |  |  |  |
| **SOX9, cytoplasmatic** | | | | | | | | | | | | |
| negative | 0 | 0% | 3 | 3% | 3 | 3% | 6 | 2% | 0.000 | 0.001 | 0.002 | 0.0223 |
| ≤50% positive | 34 | 47% | 20 | 22% | 18 | 19% | 72 | 28% |  |  |  |  |
| >50% positive | 38 | 53% | 67 | 74% | 76 | 78% | 181 | 70% |  |  |  |  |
| Total | 72 | 100% | 90 | 100% | 97 | 100% | 259 | 100% |  |  |  |  |
| **SOX9, nuclear** | | | | | | | | | | | | |
| negative | 4 | 6% | 2 | 2% | 1 | 1% | 7 | 3% | 0.015 | 0.093 | 0.003 | 0.4747 |
| ≤50% positive | 23 | 32% | 12 | 13% | 23 | 23% | 58 | 22% |  |  |  |  |
| >50% positive | 45 | 63% | 77 | 85% | 74 | 76% | 196 | 75% |  |  |  |  |
| Total | 72 | 100% | 91 | 100% | 98 | 100% | 261 | 100% |  |  |  |  |
| **SOX10** |  | | | | | | | | | | | |
| negative | 62 | 100% | 88 | 100% | 84 | 84% | 234 | 94% | 0.000 | 0.000 | 0.004 | <0.0001 |
| ≤50% positive | 0 | 0% | 0 | 0% | 3 | 3% | 3 | 1% |  |  |  |  |
| >50% positive | 0 | 0% | 0 | 0% | 13 | 13% | 13 | 5% |  |  |  |  |
| Total | 62 | 100% | 88 | 100% | 100 | 100% | 250 | 100% |  |  |  |  |
| **SLUG, cytoplasmatic** | |  | | | | | | | | | | |
| negative | 5 | 7% | 8 | 8% | 19 | 18% | 32 | 11% | 0.031 | 0.013 | 0.130 | 0.0116 |
| positive | 70 | 93% | 90 | 92% | 88 | 82% | 248 | 89% |  |  |  |  |
| Total | 75 | 100% | 98 | 100% | 107 | 100% | 280 | 100% |  |  |  |  |
| **SLUG, nuclear** | |  |  |  |  |  |  |  |  |  |  |  |
| negative | 69 | 92% | 92 | 95% | 101 | 94% | 262 | 94% | 0.715 | 0.562 | 0.419 | <0.0001 |
| ≤50% positive | 6 | 8% | 5 | 5% | 6 | 6% | 17 | 6% |  |  |  |  |
| Total | 75 | 100% | 97 | 100% | 107 | 100% | 279 | 100% |  |  |  |  |
| **CD44** |  | | | | | | | | | | | |
| negative | 27 | 39% | 58 | 65% | 69 | 70% | 154 | 60% | 0.001 | 0.000 | 0.022 | 0.0254 |
| ≤50% positive | 33 | 47% | 25 | 28% | 24 | 24% | 82 | 32% |  |  |  |  |
| >50% positive | 10 | 14% | 6 | 7% | 5 | 5% | 21 | 8% |  |  |  |  |
| Total | 70 | 100% | 89 | 100% | 98 | 100% | 257 | 100% |  |  |  |  |
| **CD24** |  |  | | | | | | | | | | |
| negative | 28 | 39% | 58 | 64% | 69 | 70% | 155 | 59% | 0.000 | 0.000 | 0.000 | 0.0062 |
| positive | 44 | 61% | 33 | 36% | 29 | 30% | 106 | 41% |  |  |  |  |
| Total | 72 | 100% | 91 | 100% | 98 | 100% | 261 | 100% |  |  |  |  |
| **TWIST, cytoplasmatic** | | | |  |  |  |  |  |  |  |  |  |
| ≤50% positive | 20 | 27% | 31 | 32% | 49 | 46% | 100 | 36% | 0.017 | 0.005 | 0.657 | 0.007 |
| >50% positive | 55 | 73% | 67 | 68% | 58 | 54% | 180 | 64% |  |  |  |  |
| Total | 75 | 100% | 98 | 100% | 107 | 100% | 280 | 100% |  |  |  |  |
| **TWIST, nuclear** | |  | | | | | | | | | | |
| negative | 16 | 21% | 46 | 48% | 67 | 64% | 129 | 47% | 0.000 | 0.000 | 0.000 | <0.0001 |
| ≤50% positive | 51 | 68% | 44 | 46% | 34 | 32% | 129 | 47% |  |  |  |  |
| >50% positive | 8 | 11% | 6 | 6% | 4 | 4% | 18 | 7% |  |  |  |  |
| Total | 75 | 100% | 96 | 100% | 105 | 100% | 276 | 100% |  |  |  |  |
