# Supplementary material for: Limited utility of tissue micro-arrays in detecting intra-tumoral heterogeneity in stem cell characteristics and tumor progression markers in breast cancer
Source: J Transl Med. 2018 May 8;16:118. doi: 10.1186/s12967-018-1495-6 (PMC5941467; doi:10.1186/s12967-018-1495-6)
Supplement: Supplementary file 4 — Additional file 4: Table S4. Conventional predictive/prognostic markers—significant correlations. [file 12967_2018_1495_MOESM4_ESM.docx]

**Additional file 4: Table S4. Conventional predictive/ prognostic markers – significant correlations**

|  | Chi-Square  (p-value) | Spearman Correlation  (p-value) | Kappa  (p-value) | Fisher's  exact test  (p-value) |
| --- | --- | --- | --- | --- |
| vs. Conventional predictive/ prognostic markers | | | | |
| ER * PR | 0.000 | 0.000 | 0.000 | <0.0001 |
| ER * HER2 IHC | 0.341 | 0.343 | 0.341 | 0.003 |
| ER * EGFR | 0.000 | 0.000 | 0.000 | 0.000 |
| ER * CK5/6 | 0.000 | 0.000 | 0.000 | <0.0001 |
| PR * EGFR | 0.004 | 0.004 | 0.004 | 0.008 |
| PR * HER2 IHC | 0.000 | 0.000 | 0.000 | <0.0001 |
| PR * CK5/6 | 0.000 | 0.000 | 0.000 | <0.0001 |
| HER2 IHC * CK5/6 | 0.011 | 0.011 | 0.011 | 0.006 |
| HER2 IHC * HER2 FISH | 0.000 | 0.000 | 0.000 | <0.0001 |
| EGFR * CK5/6 | 0.000 | 0.000 | 0.000 | <0.0001 |
| vs. Tumor progression markers | | | | |
| ER * PTEN IHC | 0.010 | 0.003 | 0.240 | 0.012 |
| ER * PTEN FISH | 0.010 | 0.159 | 0.010 | 0.1570 |
| ER * Ki-67 | 0.000 | 0.000 | 0.000 | <0.0001 |
| ER * PIK3CA IHC | 0.062 | 0.060 | 0.026 | 0.038 |
| PR * PIK3CA IHC | 0.048 | 0.049 | 0.048 | 0.071 |
| PR * Ki-67 | 0.000 | 0.000 | 0.000 | <0.0001 |
| HER2 IHC * PTEN FISH | 0.002 | 0.024 | 0.969 | <0.0001 |
| HER2 IHC * p53 | 0.000 | 0.000 | 0.000 | <0.0001 |
| HER2 IHC * Ki-67 | 0.000 | 0.000 | 0.000 | <0.0001 |
| EGFR * p53 | 0.001 | 0.001 | 0.001 | <0.0001 |
| EGFR * CK5/6 | 0.000 | 0.000 | 0.000 | <0.0001 |
| CK5/6 * PIK3CA | 0.000 | 0.000 | 0.000 | 0.001 |
| CK5/6 * p53 | 0.000 | 0.000 | 0.000 | <0.0001 |
| CK5/6 * Ki-67 | 0.000 | 0.000 | 0.000 | <0.0001 |
| vs. Stem cell markers | | | | |
| ER * mTOR | 0.003 | 0.003 | 0.003 | 0.011 |
| ER * SOX2 | 0.030 | 0.905 | 0.014 | 0.018 |
| ER * SOX9, cytoplasmatic | 0.062 | 0.060 | 0.026 | 0.038 |
| ER * SLUG, cytoplasmatic | 0.000 | 0.050 | 0.500 | <0.0001 |
| ER * SLUG, nuclear | 0.017 | 0.017 | 0.017 | 0.026 |
| ER * CD 44 | 0.028 | 0.256 | 0.008 | 0.018 |
| ER * TWIST, nuclear | 0.000 | 0.000 | 0.017 | <0.0001 |
| PR * E-Cadherin | 0.016 | 0.016 | 0.016 | 0.023 |
| PR * mTOR | 0.011 | 0.011 | 0.011 | 0.029 |
| PR * CD44 | 0.007 | 0.002 | 0.075 | 0.006 |
| PR * CD24 | 0.002 | 0.002 | 0.002 | 0.002 |
| HER2 * CD44 | 0.007 | 0.024 | 0.006 | 0.004 |
| HER2 * CD24 | 0.002 | 0.002 | 0.002 | 0.003 |
| EGFR * mTor | 0.000 | 0.000 | 0.000 | 0.003 |
| EGFR * SOX2 | 0.000 | 0.000 | 0.000 | <0.0001 |
| EGFR * SOX9, cytoplasmatic | 0.000 | 0.000 | 0.010 | <0.001 |
| EGFR * SOX9, nuclear | 0.000 | 0.000 | 0.000 | 0.001 |
| EGFR * TWIST, cytoplasmatic | 0.039 | 0.039 | 0.039 | 0.059 |
