# Supplementary material for: Limited utility of tissue micro-arrays in detecting intra-tumoral heterogeneity in stem cell characteristics and tumor progression markers in breast cancer
Source: J Transl Med. 2018 May 8;16:118. doi: 10.1186/s12967-018-1495-6 (PMC5941467; doi:10.1186/s12967-018-1495-6)
Supplement: Supplementary file 5 — Additional file 5: Table S5. Tumor progression markers—significant correlations. [file 12967_2018_1495_MOESM5_ESM.docx]

***Additional file 5:* Table S5. Tumor progression markers – significant correlations**

|  | Chi-Square  (p-value) | Spearman Correlation  (p-value) | Kappa  (p-value) | Fisher's  exact test  (p-value) |
| --- | --- | --- | --- | --- |
| vs. Tumor progression markers | | | | |
| PTEN * PIK3CA IHC | 0.005 | 0.253 | 0.001 | 0.006 |
| PIK3CA * p53 | 0.026 | 0.357 | 0.016 | 0.023 |
| vs. Stem cell markers | | | | |
| PTEN * E-Cadherin | 0.027 | 0.007 | 0.904 | 0.020 |
| PTEN * mTOR | 0.020 | 0.007 | 0.273 | 0.021 |
| PTEN * SOX2 | 0.001 | 0.000 | 0.092 | - |
| PTEN * TWIST, cytoplasmatic | 0.002 | 0.001 | 0.432 | 0.002 |
| PIK3CA * mTOR | 0.000 | 0.000 | 0.000 | 0.001 |
| PIK3CA * SLUG, cytoplasmatic | 0.006 | 0.006 | 0.006 | 0.011 |
| PIK3CA * TWIST, cytoplasmatic | 0.003 | 0.002 | 0.003 | 0.004 |
| p53 * E-Cadherin | 0.001 | 0.024 | 0.007 | <0.001 |
| p53 * mTOR | 0.013 | 0.317 | 0.004 | 0.010 |
| p53 * SLUG, cytoplasmatic | 0.027 | 0.011 | 0.563 | 0.040 |
| Ki-67 * SOX2 | 0.003 | 0.001 | 0.648 | 0.002 |
| Ki-67 * SOX10 | 0.001 | 0.000 | 0.034 | 0.000 |
| Ki-67 * CD24 | 0.000 | 0.000 | 0.000 | <0.0001 |
