# Supplementary material for: Limited utility of tissue micro-arrays in detecting intra-tumoral heterogeneity in stem cell characteristics and tumor progression markers in breast cancer
Source: J Transl Med. 2018 May 8;16:118. doi: 10.1186/s12967-018-1495-6 (PMC5941467; doi:10.1186/s12967-018-1495-6)
Supplement: Supplementary file 6 — Additional file 6: Table S6. Stem cell markers—significant correlations. [file 12967_2018_1495_MOESM6_ESM.docx]

**Additional file 6: Table S6. Stem cell markers – significant correlations**

|  | Chi-Square  (p-value) | Spearman Correlation  (p-value) | Kappa  (p-value) | Fisher's exact test  (p-value) |
| --- | --- | --- | --- | --- |
| vs. Stem cell markers | | | | |
| E-Cadherin * SOX9, cytoplasmatic | 0.001 | 0.000 | 0.006 | <0.001 |
| E-Cadherin * SOX9, nuclear | 0.005 | 0.001 | 0.026 | 0.002 |
| E-Cadherin * TWIST, nuclear | 0.015 | 0.004 | 0.023 | 0.011 |
| mTOR * TWIST, nuclear | 0.042 | 0.013 | 0.016 | 0.030 |
| SOX2 * CD44 | 0.018 | 0.001 | 0.003 | 0.054 |
| SOX2 * CD24 | 0.002 | 0.000 | 0.001 | - |
| SOX9, cytoplasmatic * CD24 | 0.063 | 0.025 | 0.043 | 0.001 |
| SOX9, cytoplasmatic * TWIST, cytoplasmatic | 0.006 | 0.048 | 0.009 | 0.055 |
| SOX9, cytoplasmatic * TWIST, nuclear | 0.033 | 0.032 | 0.053 | 0.004 |
| SLUG, cytoplasmatic * TWIST, nuclear | 0.049 | 0.016 | 0.055 | - |
| SLUG, nuclear * TWIST, nuclear | 0.036 | 0.019 | 0.117 | 0.055 |
| CD44 * TWIST, nuclear | 0.071 | 0.007 | 0.035 | - |
